# The thermal expansion of gold: point defect concentrations and pre-melting in a face-centred cubic metal

**DOI:** 10.1107/S1600576718002248

**Published:** 2018-03-26

**Authors:** Martha G. Pamato, Ian G. Wood, David P. Dobson, Simon A. Hunt, Lidunka Vočadlo

**Affiliations:** a Department of Earth Sciences, University College London, Gower Street, London WC1E 6BT, UK

**Keywords:** pre-melting phenomena, thermal expansion, gold, vacancies

## Abstract

On the basis of *ab initio* computer simulations, pre-melting phenomena have been suggested to occur in the elastic properties of hexagonal close-packed iron under the conditions of the Earth’s inner core just before melting. The extent to which these pre-melting effects might also occur in the physical properties of face-centred cubic metals has been investigated here under more experimentally accessible conditions for gold, allowing for comparison with future computer simulations of this material. The thermal expansion of gold has been determined by X-ray powder diffraction from 40 K up to the melting point (1337 K). For the entire temperature range investigated, the unit-cell volume can be represented in the following way: a second-order Grüneisen approximation to the zero-pressure volumetric equation of state, with the internal energy calculated *via* a Debye model, is used to represent the thermal expansion of the ‘perfect crystal’. Gold shows a nonlinear increase in thermal expansion that departs from this Grüneisen–Debye model prior to melting, which is probably a result of the generation of point defects over a large range of temperatures, beginning at *T*/*T*_m_ > 0.75 (a similar homologous *T* to where softening has been observed in the elastic moduli of Au). Therefore, the thermodynamic theory of point defects was used to include the additional volume of the vacancies at high temperatures (‘real crystal’), resulting in the following fitted parameters: *Q* = (*V*_0_ *K*_0_)/γ = 4.04 (1) × 10^−18^ J, *V*_0_ = 67.1671 (3) Å^3^, *b* = (*K*_0_′ − 1)/2 = 3.84 (9), θ_D_ = 182 (2) K, (*v* ^f^/Ω)exp(*s* ^f^/*k*_B_) = 1.8 (23) and *h* ^f^ = 0.9 (2) eV, where *V*_0_ is the unit-cell volume at 0 K, *K*_0_ and *K*_0_′ are the isothermal incompressibility and its first derivative with respect to pressure (evaluated at zero pressure), γ is a Grüneisen parameter, *θ*_D_ is the Debye temperature, *v* ^f^, *h* ^f^ and *s* ^f^ are the vacancy formation volume, enthalpy and entropy, respectively, Ω is the average volume per atom, and *k*_B_ is Boltzmann’s constant.

## Abstract 2

The thermal expansion of gold has been determined by X-ray powder diffraction from 40 K up to the melting point (1337 K). Gold shows a nonlinear increase in thermal expansion that departs from the associated Grüneisen–Debye model prior to melting, which has been quantified in terms of the generation of point defects.

## 1. Introduction   

Although the Earth’s inner core is recognized to be made of an iron–nickel alloy with a few percent of light elements (Birch, 1952 ▸; Allègre *et al.*, 1995 ▸; McDonough & Sun, 1995 ▸), its exact structure and composition remain unknown. Seismological models of the Earth’s inner core do not agree with mineralogical models derived from *ab initio* calculations, which predict shear-wave velocities up to 30% greater than seismically observed values (*e.g.* Vočadlo, 2007 ▸; Vočadlo *et al.*, 2009 ▸; Belonoshko *et al.*, 2007 ▸; Martorell, Brodholt *et al.*, 2013 ▸). Several proposals have been made to account for such differences, including, for instance, unusually large compositional effects, the presence of a pervasive partial melt throughout the inner core, crystal alignment, defects and grain boundaries, and anelasticity (*e.g.* Antonangeli *et al.*, 2004 ▸; Vočadlo, 2007 ▸; Belonoshko *et al.*, 2007 ▸). Another possible explanation for the observed low shear wave velocities was proposed by Martorell, Vočadlo *et al.* (2013 ▸), who reported a dramatic nonlinear reduction in the elastic constants of hexagonal close-packed (h.c.p.) iron under a hydro­static pressure of 360 GPa, just before melting (*T*/*T*_m_ > 0.96, where *T* is the temperature and *T*_m_ is the melting temperature). This was attributed to ‘pre-melting’ effects, thought to be associated with the formation of defects in the structure. Although melting is commonly classified as a first-order phase transition from solid to liquid without critical phenomena, experimental studies indicate the possibility of premonitory effects in the physical properties of a crystal at temperatures close to the melting point, so called pre-melting phenomena (Mair *et al.*, 1976 ▸). Comparison of the melting curve of iron (Sola & Alfè, 2009 ▸) with the slope of the geotherm suggests that the Earth’s entire inner core is very close to melting, with a value of *T*/*T*_m_ = 0.988 at the centre of the inner core (Martorell, Vočadlo *et al.*, 2013 ▸). Thus, the core does indeed lie in a range of *T*/*T*_m_ where the velocities might be expected to be strongly decreased near melting. Pre-melting softening has been suggested for Fe_7_C_3_, a proposed candidate component of the Earth’s inner core, just prior to melting at inner core conditions (Li *et al.*, 2016 ▸). As in the case of pure iron, the calculated sound-wave velocities agreed with seismological data; however, in the case of Fe_7_C_3_, the density was found to be too low (by ∼8%) compared to geophysical profiles (Dziewonski & Anderson, 1981 ▸).

To date, the computer calculations reported by Martorell, Vočadlo *et al.* (2013 ▸) and Li *et al.* (2016 ▸) are the only results on pre-melting of a metal that are directly applicable to the Earth’s inner core, although computer simulations of another h.c.p. metal, Mg, have also suggested that pronounced changes in density and elastic moduli occur just prior to melting at *T*/*T*_m_ > ∼0.97 (Bavli, 2009 ▸; Bavli *et al.*, 2011 ▸). In the context of planetary cores, it is therefore essential to systematically investigate such phenomena not only for a range of pressures and temperatures (*P*−*T*) at inner core conditions, but also for iron alloyed with both nickel and light elements in a multicomponent system. However, measuring the pressure dependence of pre-melting effects at such conditions and to the required precision is extremely challenging. Pre-melting effects have been observed or are suggested to occur in a range of physical properties in other metals. For example, the shear modulus of tin has been experimentally shown to decrease by more than 50% at temperatures within about 1% of its melting point (Nadal & Le Poac, 2003 ▸). Also, experiments have shown enhanced temperature dependence close to melting in the elastic modulus (*C*_44_) of aluminium (Gordon & Granato, 2004 ▸, and references therein), in the electrical conductivities of lead (Pokorny & Grimvall, 1984 ▸) and of iron (Secco & Schloessin, 1989 ▸), and in the thermal expansion of sodium single crystals (Adlhart *et al.*, 1974 ▸).

In light of this, we aim to investigate to what extent pre-melting behaviour may occur in the physical properties of metals by a combination of *ab initio* computer simulations and experiments at accessible conditions. We also wish to determine to what extent changes in elastic moduli with temperature are correlated with changes in the unit-cell parameters, since the latter are more easily measured as a function of pressure and temperature. Here, we report precise measurements at atmospheric pressure of the unit-cell parameters and the thermal expansion coefficient of a face-centred cubic (f.c.c.) metal, pure gold, from low temperatures (40 K) up to the melting point, approaching *T*_m_ in much finer temperature steps than have been reported previously (Simmons & Balluffi, 1962 ▸; Touloukian *et al.*, 1975 ▸). Gold is an ideal test material since it crystallizes in a simple monatomic (*i.e.* with one atom in the primitive unit cell) f.c.c. structure (space group *Fm* 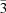 *m*); it is chemically inert and has a relatively low melting temperature (1337.33 K; Hieu & Ha, 2013 ▸). The pressure–volume–temperature equation of state for gold has been extensively studied and several equation of state (EoS) models have been proposed (*e.g.* Anderson *et al.*, 1989 ▸; Jamieson *et al.*, 1982 ▸; Tsuchiya, 2003 ▸; Heinz & Jeanloz, 1984 ▸; Shim *et al.*, 2002 ▸; Yokoo *et al.*, 2009 ▸). Furthermore, pre-melting effects have been suggested to occur in the elastic properties of noble metals, such as gold and palladium, which exhibit large departures from linearity at elevated temperatures (Yoshihara *et al.*, 1987 ▸; Collard & McLellan, 1991 ▸).

Precise measurements of the temperature dependence of the lattice parameter of Au, up to melting, have been made by Simmons & Balluffi (1962 ▸) during their determination of the equilibrium concentration of vacancies in Au by differential dilatometry. In this method (*e.g.* Siegel, 1978 ▸; Wollenberger, 1996 ▸; Kraftmakher, 1998 ▸), the relative changes in the bulk volume of a crystalline sample (Δ*V*_B_/*V*_B_) and also in its unit-cell volume (Δ*V*_C_/*V*_C_) are determined, ideally from the same specimen under exactly the same temperature conditions. Since the number of atoms in the sample must be conserved, it can be readily shown that the vacancy concentration, *N*_vac_ */N*_atoms_, is given by (Δ*V*_B_/*V*_B_) – (Δ*V*_C_/*V*_C_). Differential dilatometry thus provides an absolute method for the determination of vacancy concentrations, but such experiments are not without their difficulties, in particular with regards to the measurement of Δ*V*_B_/*V*_B_ to the required accuracy. Large specimens are generally required; for example, Simmons & Balluffi (1962 ▸) employed a gold bar of 99.999% purity and size 12.7 × 12.7 × 500 mm to measure the relative change in its length, Δ*L*/*L*, to ∼1 × 10^−5^. Such measurements would be extremely difficult to perform sufficiently well at high pressure.

Because of the experimental difficulties inherent in differential dilatometry, an alternative approach to determining the formation parameters of thermally induced defects is to do this *via* a detailed analysis of the temperature dependence of the thermal expansion of the material at high temperatures. In some of the earliest work in this area, Lawson (1950 ▸) proposed that the anomalous thermal expansion observed in a number of substances (especially AgBr and AgCl) just below their melting points was the result of pre-melting phenomena associated with thermally generated defects. For NaCl, Merriam *et al.* (1962 ▸) assumed that the high-temperature thermal expansion was governed by two terms: a ‘normal’ component (assumed to vary linearly with temperature) and an ‘anomalous’ component from the thermally generated defects, which increased exponentially with temperature. More recently, Wang and Reeber have applied a similar though more elaborate approach to ionic crystals (Wang & Reeber, 1994 ▸) and to both body-centred cubic (Wang & Reeber, 1998 ▸) and f.c.c. metals (Wang & Reeber, 2000 ▸). In this method, described in detail in §3.4, the behaviour of the unit-cell volume of the ‘real’ crystal is modelled in terms of that expected from a ‘perfect’ (*i.e.* defect-free) crystal, modified by a term describing the contribution to the thermal expansion from thermally induced vacancies. Provided that the assumptions inherent in determining the properties of the ‘perfect’ crystal are valid, this method therefore provides a route whereby quantities such as the vacancy formation enthalpy can be determined from a single set of thermal expansion measurements.

## 2. Methods   

For a successful determination of any pre-melting effects on the density and thermal expansion of Au we need to measure precisely the unit-cell parameters up to the melting point, with fine temperature spacing as the melting point is approached. The measurements (on Au from ESPI Metals, 99.999% purity) were performed using a PANalytical X’Pert Pro powder diffractometer. This instrument, operating in Bragg–Brentano parafocusing reflection geometry, is equipped with an incident beam Ge(111) Johansson geometry focusing monochromator, producing a Co *K*α_1_ incident beam, and can be fitted with environmental stages covering the range from 40 to 1373 K (with a readily achievable temperature resolution of 1 K). The X-ray tube was operated at 40 kV and 30 mA. In the incident and diffracted beam optics, variable width divergence and anti-scatter slits were used, together with a 10 mm wide beam mask in the incident beam, in order to illuminate a constant 10 × 8.5 mm area of the sample; 0.04 radian Soller slits were present in both the incident and diffracted beams to reduce the axial divergences and an ‘X’Celerator’ position-sensitive detector was used. Data collections were performed over a 2*θ* range of 40–151° below 298 K and 40–154° above 298 K, with collection times of ∼105 min and ∼55 min at each temperature, respectively. After the experiments reported here, the zero 2*θ* angle of the diffractometer was determined using an Si standard (NBS SRM640). Diffraction data between 40 and 300 K were obtained using an Oxford Cryosystems PheniX-FL low-temperature stage (a modified version of the standard PheniX stage; Wood *et al.*, 2018 ▸), and data between 298 and 1373 K were collected using an Anton Paar HTK1200N heated stage. The powdered Au sample was dispersed on top of MgO (Aldrich 99.99%), which helps to constrain the specimen displacement and 2*θ* offset in the Rietveld refinements and also provides a reservoir for Au when it melts. The MgO was fired overnight at 1073 K in air before use. Before the X-ray measurements were collected, the sample (MgO + Au dispersed across the top) was annealed for 300 min at 773 K in the diffractometer (in the Anton Paar HTK1200N heated stage) in order to reduce the full width at half-maximum of the diffraction peaks from Au.

Below 298 K, in the PheniX-FL cold stage, the sample was held in helium exchange gas at atmospheric pressure. The sample was initially cooled at 2 K min^−1^ to 80 K and then at 1 K min^−1^ to 40 K; subsequent increases in temperature were made manually at 2 K min^−1^ and the sample was then allowed to equilibrate for at least 10 min before the diffraction patterns were collected. Measurements were made on warming at intervals of 10 K from 40 to 200 K and intervals of 20 K from 200 to 300 K. Above 298 K, measurements were performed in air and the sample was heated at 5 K min^−1^, after which it was equilibrated for a time which varied from 25 min (at 323 K) to 6 min (at 413 K) and above. Measurements were made at intervals of 20 K between 200 and 1273 K, 10 K between 1273 and 1313 K, and 2 K up to the point where all of the gold was molten (1339 K). The temperature control was better than ±0.1 K throughout the entire analysis. The intensities of the diffraction patterns were converted from variable to fixed divergence slit geometry using the software supplied by the manufacturer, after which the data were analysed by Rietveld refinement using the *GSAS* suite of programmes (Larson & Von Dreele, 2000 ▸; Toby, 2001 ▸). In addition to the cell parameters of Au and MgO, the isotropic atomic displacement parameters, scale factor, sample shift and profile shape parameters were varied during the fitting procedure. In total, 23 variables were included in the refinement, with ∼6660 data points in each diffraction pattern, the effective step size being ∼0.017° in 2*θ.*

Owing to a small offset between the data collected using the high- and low-temperature stages, the high-temperature results were scaled to match the low-temperature volumes, by minimizing the residuals of a second-order polynomial passing through the 200–280 K (cold-stage) and 313–393 K (hot-stage) data. The resulting scaling factor used for the high-temperature data is 0.99984. This scaling has minimal effect on the fitted values of the variable parameters in the models used to describe the thermal expansion.

## 3. Results and discussion   

### 3.1. Lattice parameters of gold as a function of temperature   

**Figure 1:**
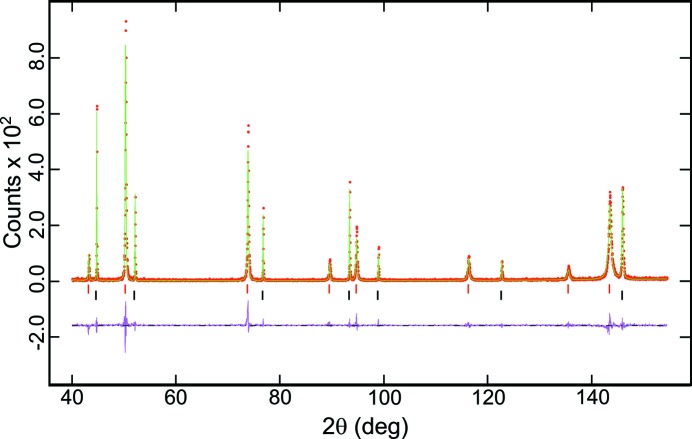
X-ray powder diffraction pattern of gold (+ MgO) at 298 K, collected with the sample in the hot stage. Observed (red points) and calculated patterns (green line) and their differences (purple lower trace) are also shown. The tick markers show the position of the Bragg reflections from top down: MgO (red) and Au (black).

A typical diffraction pattern of Au, collected at room temperature, is presented in Fig. 1 ▸.

**Figure 2:**
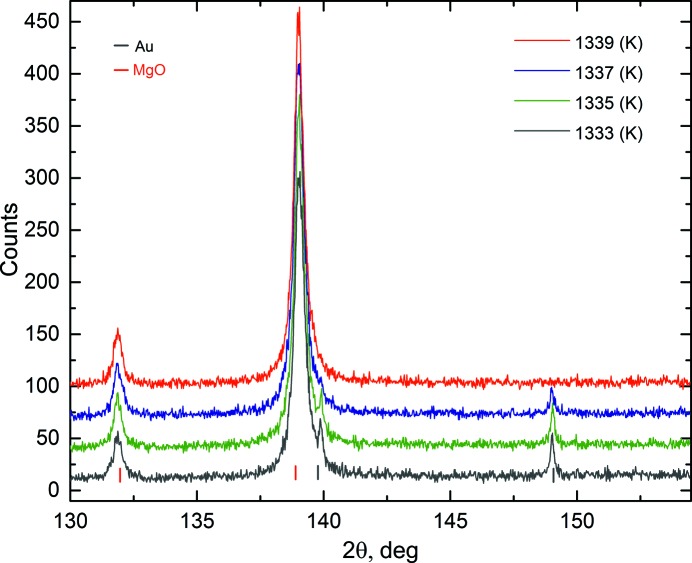
X-ray powder diffraction patterns of gold (black markers) and MgO (red markers) at high 2θ angles, at different temperatures approaching melting. Note that the gold peaks disappear at 1339 K, indicating that gold melted between 1337 and 1339 K, in perfect agreement with the melting temperature reported in the literature.

Au diffraction patterns at high 2θ angles and at four different temperatures approaching melting are shown in Fig. 2 ▸. Au peaks are present in the diffraction pattern at 1337 K but disappear at 1339 K, indicating that the gold melted between 1337 and 1339 K. This is in perfect agreement with the melting temperature reported in the literature (1337.33 K; Hieu & Ha, 2013 ▸), demonstrating the accuracy of the heating stage thermometry. Given the very small amount of Au still present at 1337 K (Fig. 2 ▸), we did not include this point in the subsequent analysis of the data.

**Figure 3:**
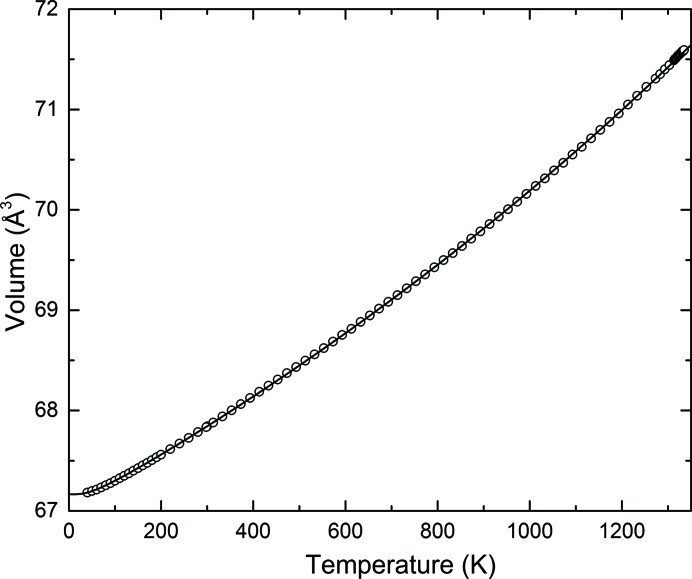
Unit-cell volume of gold as a function of temperature showing the entire temperature range. The error bars are smaller than the symbols. The solid line represents the fit of the data to a second-order Grüneisen approximation to the zero-pressure equation of state [equation (3)].

The evolution of the unit-cell volume of gold as a function of temperature is reported in Fig. 3 ▸, and the lattice parameters and unit-cell volumes are tabulated in Table S1. Our results appear to be in excellent agreement with those of Simmons & Balluffi (1962 ▸) for gold of nominally the same purity; for example, at 1333 K the change in lattice parameters relative to its value at 293 K found in the present study (0.018153) corresponds exactly to that tabulated by Simmons & Balluffi (0.01815).

### 3.2. A simple model of the thermal expansion above room temperature   

The high-temperature behaviour of gold was modelled using several approaches. The temperature evolution of the volume above room temperature can be expressed, according to Fei (1995 ▸), as
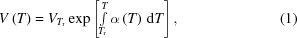
where 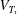 is the volume at a chosen reference temperature *T*_r_ (300 K in this case) and 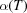 is the volumetric thermal expansion coefficient, given by a linear expression of the form
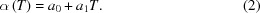
The resulting values for the data from 298 to 1335 K, fitted in *EoSFit7* (Angel *et al.*, 2014 ▸), are 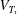 = 67.854 (2) Å^3^, *a*_0_ = 3.62 (2) × 10^−5^ K^−1^ and *a*_1_ = 1.88 (3) × 10^−8^ K^−2^ and are in fairly good agreement with the values 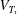 = 67.85 Å^3^ (fixed), *a*_0_ = 3.179 (139) × 10^−5^ K^−1^ and *a*_1_ = 1.477 (310) × 10^−8^ K^−2^ reported by Hirose *et al.* (2008 ▸). The volumetric thermal expansion coefficient, 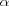, at ambient conditions (300 K and 1 bar; 1 bar = 10^5^ Pa) is predicted to be 4.18 (2) × 10^−5^ K^−1^ using equation (2), and is higher than that reported by Hirose *et al.* (2008 ▸) (3.62 × 10^−5^ K^−1^), but is in excellent agreement with the value quoted by Simmons & Balluffi (1962 ▸) (equivalent to 4.17 × 10^−5^ K^−1^ for the volumetric expansion coefficient), and is in good agreement with other values determined both experimentally (4.28 × 10^−5^ K^−1^; Touloukian *et al.*, 1975 ▸; Anderson *et al.*, 1989 ▸) and from theoretical calculations (4.52 × 10^−5^ K^−1^; Tsuchiya, 2003 ▸). At 1335 K, the expansion coefficient on this model is 6.13 (2) × 10^−5^ K^−1^.

### 3.3. Grüneisen–Debye models of thermal expansion   

A more physically meaningful parameterization of experimental *V*(*T*) dependency, covering the entire temperature range, can be obtained using the Grüneisen approximations for the zero-pressure equation of state, in which the effects of thermal expansion are considered to be equivalent to elastic strain induced by thermal pressure (*e.g.* Wallace, 1998 ▸). This approach allows investigation of the dynamics of the material by enabling evaluation of the Debye temperature. The second-order approximation, derived on the basis of a Taylor series expansion of (*PV*) to second order in Δ*V*, is commonly reported as being more appropriate for the fitting and extrapolation of higher-temperature data (Vočadlo *et al.*, 2002 ▸; Lindsay-Scott *et al.*, 2007 ▸; Trots *et al.*, 2012 ▸; Hunt *et al.*, 2017 ▸) and takes the form (Wallace, 1998 ▸)
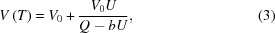
where
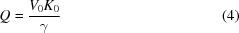
and
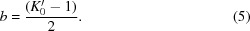
In equations (3)–(5) [Disp-formula fd5], *V*_0_ is the hypothetical volume at *T* = 0 K, γ is a Grüneisen parameter, assumed to be pressure and temperature independent, and 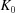 is the isothermal incompressibility at *T* = 0 K and *P* = 0 GPa. 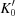 is its first pressure derivative, also evaluated at *T* = 0 K and *P* = 0 GPa.

In cases when the behaviour of *V*(*T*) is more complex, a third-order Grüneisen approximation (see Wood *et al.*, 2004 ▸) can be employed, which takes the form
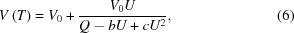
where
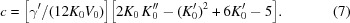


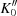
 is the second derivative of the isothermal incompressibility with respect to pressure, at *T* = 0 K and *P* = 0 GPa.

The internal energy, *U*(*T*), required in equations (3) and (6) can be calculated using the Debye model to describe the energy of thermal vibrations:
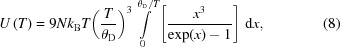
where *N* is the number of atoms in the unit cell (in this case *N* = 4), *k*_B_ is Boltzmann’s constant and θ_D_ is the Debye temperature.

The solid line in Fig. 3 ▸ shows the result obtained from fitting equation (3) to the data by weighted nonlinear least squares, with resulting values for the four fitted constants of *Q* = 4.110 (8) × 10^−18^ J, *V*_0_ = 67.1657 (4) Å^3^, *b* = 4.28 (4) and *θ*_D_ = 173 (2) K.

The Debye temperature of Au determined from equation (3), 173 (2) K, is in very good agreement with values reported in the literature, which range from 165 to 170 K, from calorimetric data and derived from elastic measurements (*e.g.* Neighbours & Alers, 1958 ▸, and references therein; Anderson *et al.*, 1989 ▸).

An estimate of the first pressure derivative of the incompressibility 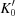 can be obtained directly from the coefficient *b* in equation (3) (see Table 1 ▸). The resulting value, 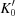 = 9.57 (8), is, however, higher than published values for 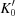, which range between 5 and 6.2 (Anderson *et al.*, 1989 ▸; Jamieson *et al.*, 1982 ▸; Tsuchiya, 2003 ▸; Heinz & Jeanloz, 1984 ▸; Shim *et al.*, 2002 ▸; Yokoo *et al.*, 2009 ▸). The incompressibility itself can also be estimated from equation (3), provided the Grüneisen parameter is known. Grüneisen parameter values reported in the literature are between 2.95 and 3.215 (Anderson *et al.*, 1989 ▸; Jamieson *et al.*, 1982 ▸; Tsuchiya, 2003 ▸; Heinz & Jeanloz, 1984 ▸; Shim *et al.*, 2002 ▸; Yokoo *et al.*, 2009 ▸). If we apply the minimum value of *γ* (2.95) in the present case, we obtain a value of 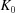 = 180.5 (4) GPa, which is in very good agreement with the values of 180 GPa at 0 K and 167.5 GPa at 300 K reported by Yokoo *et al.* (2009 ▸), whereas using the maximum value of 3.215 we obtain 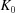 = 196.7 (4) GPa.

**Figure 4:**
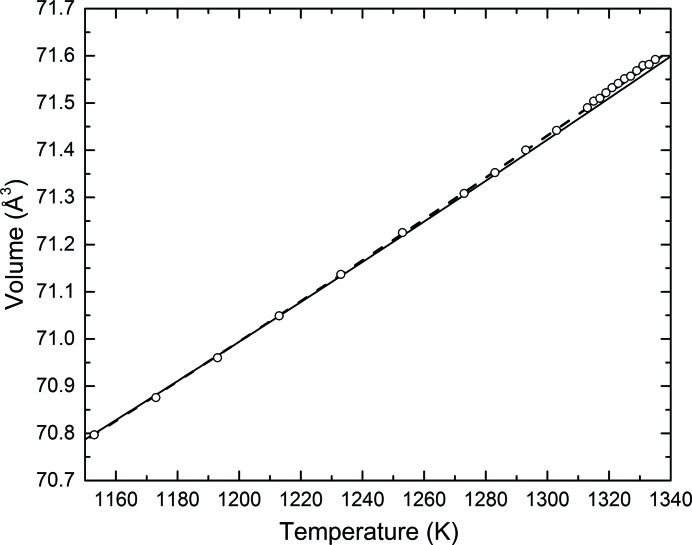
Unit-cell volumes of gold in the high-temperature region expanded to show the possible pre-melting zone. The error bars are smaller than the symbols. The solid and dashed lines represent the fits of the data to second-order and third-order Grüneisen approximations to the zero-pressure equation of state [equations (3) and (6)].

Although equation (3) provides a good basis within which to assess the behaviour of the material, the theory suffers from several deficiencies. In particular, a harmonic approximation is used to calculate *U*(T). Also, a limitation of the approach is that *Q* and *b* are assumed to be temperature independent, whereas in reality the Grüneisen parameter has some temperature dependence (*e.g.* Vočadlo *et al.*, 2002 ▸). These deficiencies in the model can be reflected in the fitted values of the four parameters, which should therefore be treated carefully. However, it can be seen that equation (3) provides an excellent fit up to about 1200 K, above which point the calculated curve is not sufficiently steep (see Fig. 4 ▸). In an effort to improve the fit we tried including the electronic contribution to the heat capacity, *C*_el_, using a linear term for *C*_el_(*T*); however, this led to an essentially identical fit and did not improve the agreement at high temperatures. The deviation from the model at high temperatures may arise from the failure of the harmonic Debye approximation as anharmonicity becomes progressively more important (Vočadlo *et al.*, 2002 ▸) or may arise from other contributions to the heat capacity, such as the formation of defects; these possibilities are discussed below.

To account for possible anharmonicity and for the insufficient curvature in the region immediately below melting (*T*_m_), we also employed a third-order Grüneisen approximation (see Wood *et al.*, 2004 ▸) [equation (6)]. For the purpose of comparison, and in order to examine the most suitable method for describing the behaviour of the perfect crystal of Au at high temperatures, the values of the fitted variable parameters in the various equations employed are reported in Table 1 ▸.

**Figure 5:**
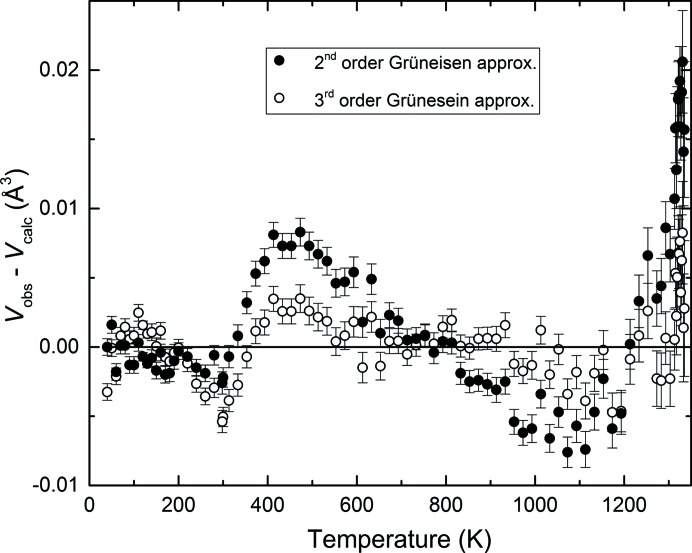
Differences between measured and calculated volumes as a function of temperature, when employing a second-order (filled symbols) or a third-order (open symbols) Grüneisen approximation [equations (3) and (6)].

The differences between observed and calculated volumes, employing either the second- or third-order Grüneisen approximations [equations (3) and (6)], are also displayed in Fig. 5 ▸.

The third-order approximation appears to provide a better fit of the data up to 1300 K, where the maximum volume residuals are less than 0.005 Å^3^, as opposed to the second-order approximation which displays deviations up to 0.01 Å^3^ (Fig. 5 ▸). At temperatures close to melting, however, both models fail to represent the observed data, where a distinct systematic deviation of up to 0.02 and 0.01 Å^3^ (twice the maximum deviations below 1300 K) is observed for the second- and third-order approximations, respectively.

By fitting equation (6) to the data for *V*(*T*) we obtained values of the constants *Q* = 3.97 (2) × 10^−18^ J, *V*_0_ = 67.1678 (3) Å^3^, *b* = 2.8 (2), *θ*_D_ = 188 (2) K and *c* = −0.44 (5) × 10^19^ J^−1^. Although the third-order Grüneisen approximation appears to provide a better fit to the data (see Figs. 4 ▸ and 5 ▸), a Debye temperature of 188 (2) K is obtained through this approach, which is high compared to the more commonly reported values of 165–170 K (Anderson *et al.*, 1989 ▸; Jamieson *et al.*, 1982 ▸; Tsuchiya, 2003 ▸; Heinz & Jeanloz, 1984 ▸; Shim *et al.*, 2002 ▸; Yokoo *et al.*, 2009 ▸); if the parameter *b* in equation (6) is fixed at 3/2, corresponding to a value of 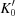 = 4, an even higher Debye temperature of 216 (2) K is obtained.

The limitations of equation (6) are also reflected in the numerically large fitted value of the parameter *c* = −0.44 (5) × 10^19^ J^−1^; by substituting the values of 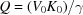 and 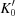 = 6.6 [from equation (5)] in the relationship 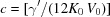 × 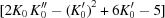 we obtain 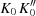 = −100 (12). Even if we fix 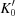 = 4, we obtain 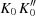 = −188 (2), which is much higher than the value resulting from the implied values of 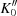 in many of the commonly used isothermal equations of state for Au. For example, by fitting a third-order Birch–Murnaghan equation to the data reported by Hirose *et al.* (2008 ▸) we obtained 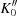 = −0.04, which corresponds to 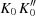 = −6.5 (fixing 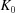 at 167 GPa). In particular, our resulting 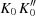 = −188 (2) value is much higher than that required by the third-order Birch–Murnaghan equation, where 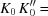 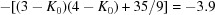 for 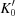 = 4 (*e.g.* Angel, 2000 ▸). Thus, although Fig. 5 ▸ appears to show that the third-order Grüneisen approximation does a better job in fitting the data, analysis of the fitted parameters above gives a contrary conclusion, *i.e.* the fitted parameters give a poorer representation of the true material properties.

### 3.4. The effect of thermally induced vacancies on the thermal expansion   

At very high homologous temperatures, a further disadvantage of the Grüneisen–Debye model discussed above is the fact that it does not consider the presence of thermally generated defects in a material. These defects can produce a significant contribution to the thermal expansion, especially as the temperature approaches the melting point where the defect concentration is greatest (*e.g.* Lawson, 1950 ▸; Merriam *et al.*, 1962 ▸; Simmons & Balluffi, 1962 ▸; Gilder & Wallmark, 1969 ▸; Siegel, 1978 ▸; Wollenberger, 1996 ▸; Kraftmakher, 1998 ▸). Although in some circumstances the formation of interstitials has been found to have a significant effect on the physical properties of f.c.c. metals (*e.g.* Gordon & Granato, 2004 ▸), in our estimation of the contribution from thermally induced defects to the thermal expansion of Au, we have assumed that only the formation of monovacancies is significant, as it is generally considered that in simple close-packed structures the formation of vacancies will dominate over formation of interstitials as the energy required is much lower (*e.g.* Kraftmakher, 1998 ▸; Simmons & Balluffi, 1962 ▸).

The thermal expansion of a crystal at high temperature can therefore be treated as having contributions both from the perfect crystal and from its thermal defects. Here we use the formalism reported by Wang & Reeber (2000 ▸) whereby a quasi-harmonic model [in this case the Grüneisen–Debye model employed earlier, equation (3), rather than the summation over Einstein oscillators used by Wang and Reeber] represents the thermal expansion of the perfect crystal, and the contributions from lattice defects at high temperature (here termed the real crystal) are described by the thermodynamic theory of point defects. The expected volume contribution from such defects is expressed as follows:
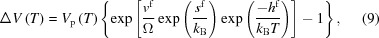
where *V*_p_(*T*) is the volume of a perfect crystal, 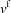, 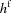 and 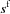 are the formation volume, enthalpy and entropy of the point defect, respectively, 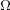 is the average volume per atom, and 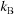 is Boltzmann’s constant; 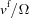 and 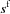 are assumed to be constants.

If we use the Grüneisen approximations for the zero-pressure equation of state with the Debye approximation of the internal energy [equations (3)–(5) [Disp-formula fd5]] to describe the volume for a perfect crystal, *V*_p_(*T*), the volume of the real crystal is
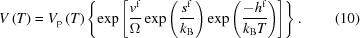
In fitting equation (10) to the data for *V*(T) we obtained *Q* = 4.04 (1) × 10^−18^ J, *V*_0_ = 67.1671 (3) Å^3^, *b* = 3.84 (9), *θ*_D_ = 182 (2) K, 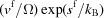 = 1.8 (23) and 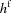 = 0.9 (2) eV. The enthalpy of formation obtained matches the values reported in the literature, which range from 0.6 to 0.962 eV (see Wollenberger, 1996 ▸; Kraftmakher, 1998 ▸, and references therein). The incompressibility and its pressure derivative, estimated from equations (3) and (4), assuming *γ* = 2.95, resulted in the following values: 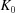 = 177.5 (5) GPa and 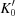 = 8.7 (2).

**Figure 6:**
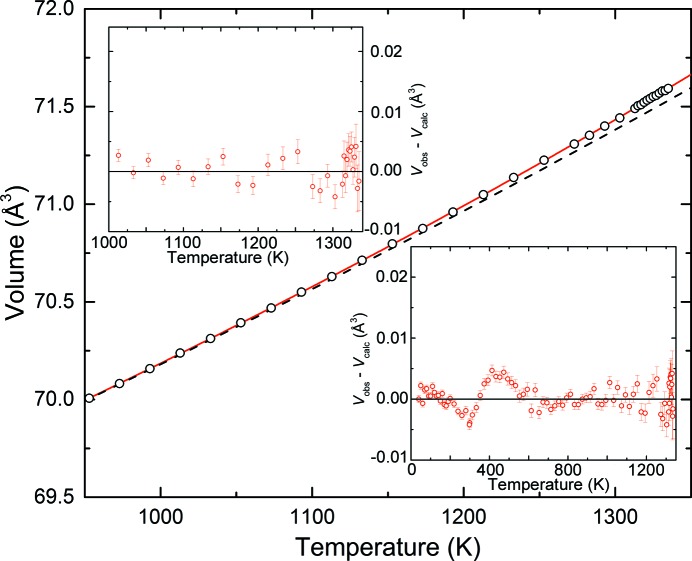
Unit-cell volume of gold expanded in the high-temperature region to show the possible pre-melting region. The error bars are within the symbols. The solid red line represents the fit of the data to equation (10) (real crystal) and the dashed line is the perfect crystal component [*i.e. V*_p_(*T*)]. Differences between measured and calculated volumes including the defect contribution to the volume of the real crystal as a function of temperature are shown in the insets.

The observed and calculated volumes and their differences, taking into account the contribution from lattice defects, are displayed in Fig. 6 ▸. An excellent fit to the data over the entire range, particularly near melting, is clearly visible, with maximum volume residuals less than 0.005 Å^3^. In particular, at temperatures above 600 K the residuals are not systematic with temperature, unlike those displayed when using the second- or third-order Grüneisen approximations (see Fig. 5 ▸).

**Figure 7:**
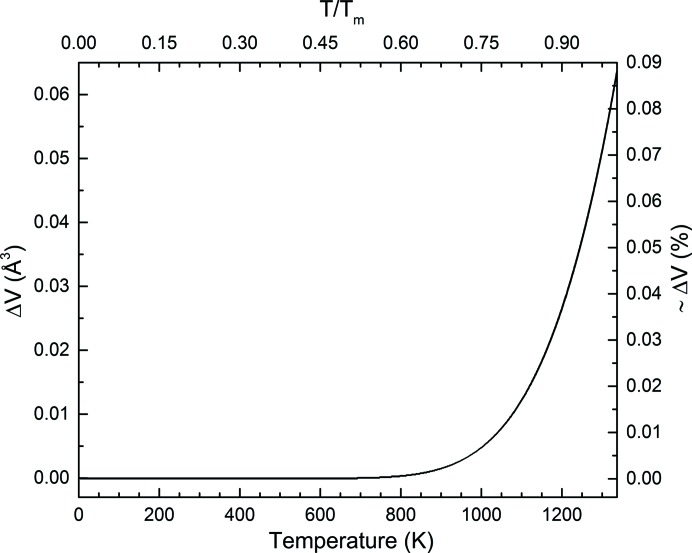
Difference in volume (Δ*V*) between a real and a perfect Au crystal [equation (9)] as a function of temperature (bottom *x* axis) and homologous temperature, *T*/*T*_m_ (top *x* axis). The difference in volume is also reported as a percentage on the right.

The volume difference between the real and perfect Au crystals [*i.e.* the difference in the fitted values of equations (10) and (3)] is reported in Fig. 7 ▸. The presence of defects in Au has a clear effect above 800 K, and the difference between the real and perfect crystals reaches almost 0.1% at *T*_m_. Although this difference in volume is minor, it should be noted that Δ*V* becomes significant at roughly the same temperature where deviations from linearity were observed in the elastic properties of gold (Collard & McLellan, 1991 ▸).

**Figure 8:**
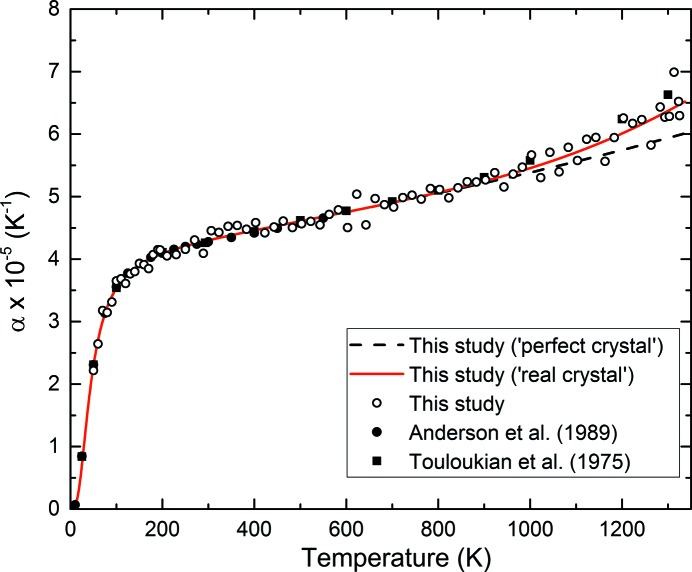
Volumetric thermal expansion coefficient of gold as a function of temperature. The open data points were obtained by numerical differentiation of the data reported in Table S1 and Fig. 2 ▸ [equation (11)], selecting a 20 K window (see text). The red solid line represents the calculated real crystal model [equation (10)] and the dashed black line is the perfect crystal component thereof [*i.e. V*_p_(*T*), see text for details]. Measured values reported in the literature (filled symbols) are also plotted for comparison.

The quality of fit of the model to the measured data is also reflected in the thermal expansion. Fig. 8 ▸ shows the volumetric thermal expansion coefficient of Au obtained from
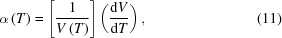
where *T* is the temperature and d*V*/d*T* is the rate of volume change at *T*. Given the variable spacing of the data points (20–2 K spacing, see Table S1), we chose a fixed window of 20 K, through which we fitted a first- or second-order polynomial to derive the experimental values. The thermal expansion is reported at the mid-point of the window. The order of the polynomial was 1 when the data spacing was 20 K; otherwise it was 2.

It can be seen that the fit resulting from equation (10) corresponds well to the data points shown in the figure, whereas equation (3) systematically underestimates the expansion coefficient above ∼800 K.

**Figure 9:**
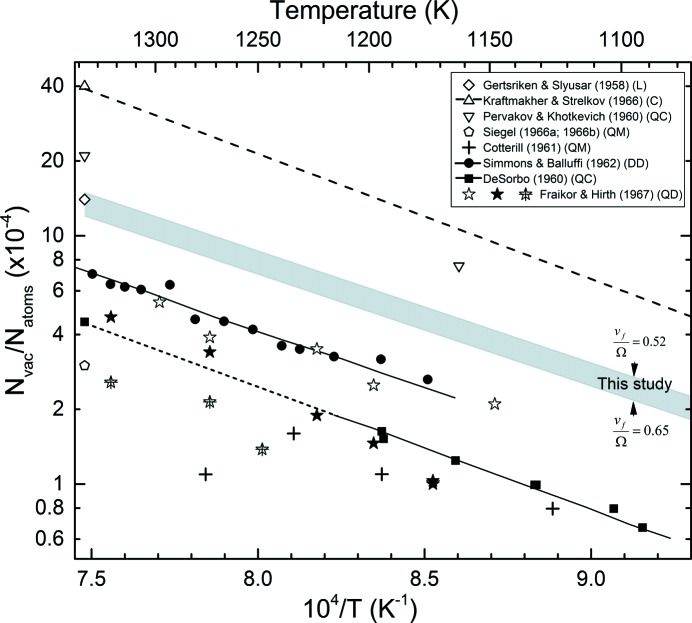
Temperature dependence of vacancy concentrations in gold, determined by a variety of methods. The letters indicate that the vacancy concentration was determined from differential dilatometry (DD; Simmons & Balluffi, 1962 ▸); calorimetry of quenched samples (QC; DeSorbo, 1960 ▸; Pervakov & Khotkevich, 1960 ▸); linear extrapolation of thermal expansivity (L; Gertsriken & Slyusar, 1958 ▸); specific heat (C; Kraftmakher & Strelkov, 1966 ▸); dilatometry of quenched samples (QD; Fraikor & Hirth, 1967 ▸); electron microscopy of quenched samples (QM; Cotterill, 1961 ▸; Siegel, 1966*a* ▸,*b* ▸).

Au is considered to be one of the most favourable metals for studies of vacancy formation. The equilibrium vacancy concentration at the melting point, as determined previously, ranges from 7 × 10^−4^ (differential dilatometry) to 4 × 10^−3^ (specific heat) – see *e.g.* Table 11 of Kraftmakher (1998 ▸). Siegel (1978 ▸) reported an equilibrium total vacancy concentration at the melting temperature of 7 × 10^−4^, with a monovacancy formation enthalpy of 0.94 eV and a monovacancy formation entropy of 0.7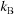. In fitting the data for *V*(*T*) to equation (10) we obtained 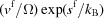 = 1.8 (23) and 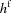 = 0.9 (2) eV. To calculate the vacancy concentration from our results it is then necessary to assume a value for 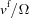, the ratio of the vacancy formation volume to the atomic volume. Emrick (1980 ▸) quotes a value of 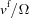 = 0.52 for close to room temperature but suggests that this might rise to 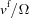 = 0.65 at higher temperatures, a value that is also given by Seeger (1973 ▸). For 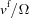 = 0.52 and 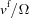 = 0.65 we obtain values of 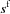 = 1.26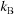 and 1.03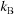, respectively*.* With these values, we can calculate the vacancy concentration from (Wollenberger, 1996 ▸)
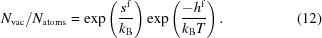
At the melting temperature, we obtain 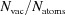 = 1.5 × 10^−3^ (for 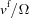 = 0.52) and 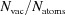 = 1.2 × 10^−3^ (for 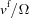 = 0.65), which is in fair agreement with the results of Simmons & Balluffi (1962 ▸) from differential dilatometry [7.2 (6) × 10^−4^] and within the range of values for gold compiled by Kraftmakher (1998 ▸), determined by a variety of methods (7 × 10^−4^ to 40 × 10^−4^). The expected temperature dependence of the vacancy concentration as determined in the present study and found previously by a variety of methods is shown in Fig. 9 ▸.

### 3.5. Thermal expansion and heat capacity of gold   

A further check on the reliability of our thermal expansion model is provided by considering the relationship between thermal expansion and heat capacity.

The volumetric or isochoric heat capacity (*C*_V_) is the change in internal energy with temperature, at constant volume (Poirier, 2000 ▸):
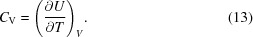
The assumption of the Debye model for internal energy [equation (8)] therefore gives a molar heat capacity of
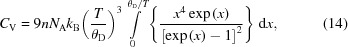
where *n* is the number of atoms per formula unit and *N*_A_ is Avogadro’s number. Experimental measurements of heat capacity are usually made at constant pressure and the isobaric heat capacity (*C*_P_) is related to the isochoric heat capacity by
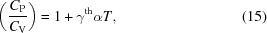
where γ^th^ is the thermal Grüneisen parameter. In general, the heat capacity of a system can be represented as the sum of various contributions (Safonova *et al.*, 2016 ▸):
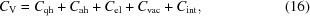
where *C*_qh_ is the Debye heat capacity [equation (14)], *C*_ah_ is the contribution to the heat capacity arising from the anharmonicity of vibrational motion of atoms, *C*_el_ is the electronic heat capacity, and *C*_vac_ and *C*_int_ are contributions from equilibrium point defects, namely vacancies and interstitial atoms, respectively.

In their estimation of contributions to the specific heat from lattice defects, Cordoba & Brooks (1971 ▸) assumed that only the formation of monovacancies and divacancies is significant; the contribution to *C* from the monovacancies is given by
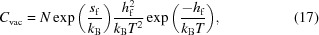
where 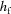 is the enthalpy of formation for a monovacancy and 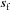 is the entropy of formation (Cordoba & Brooks, 1971 ▸).

In our analysis we did not include the electronic contribution to the specific heat as it is small and did not improve the fit at high temperatures. Balcerzak *et al.* (2014 ▸) reported that the electronic contribution amounts to 0.3% and up to 3.5% of the total specific heat at 100 and 1300 K, respectively, but according to Cordoba & Brooks (1971 ▸) the electronic contribution is only 0.1% at 1330 K.

Anharmonicity is usually considered as a plausible reason for the nonlinear increase in high-temperature specific heat and thermal expansion of metals. However, almost all theoretical calculations of the anharmonicity predict these contributions to be approximately linear. Therefore, it seems unlikely that a nonlinear anharmonicity contribution to the specific heat is much larger than the linear term (Kraftmakher, 1998 ▸). According to Cordoba & Brooks (1971 ▸), the contribution to the heat capacity from anharmonic lattice vibrations only becomes significant at temperatures considerably above the Debye temperature, where the anharmonic contribution appears to be positive. However, these authors concluded that, within the uncertainties in the parameters used in calculating the excess heat capacity, the contribution could be zero or slightly negative.

For our measurements, the volumetric thermal expansion coefficient (α), Debye temperature (*θ*_D_) and thus *C*_V_ are obtained directly from the fit of equation (10) to the data. We assume the Grüneisen parameter in equation (4) (γ) and the thermal Grüneisen parameter (γ^th^) to be the same.

**Figure 10:**
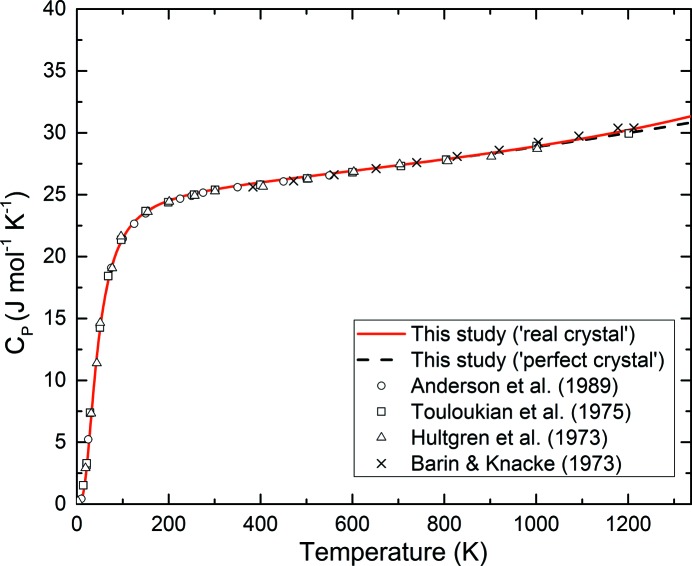
Calculated isobaric heat capacity of gold at ambient pressure. Measured values reported in the literature are also plotted for comparison: open circles from Anderson *et al.* (1989 ▸); crosses from Barin & Knacke (1973 ▸) as reported by Yokoo *et al.* (2009 ▸); open triangles from Hultgren *et al.* (1973 ▸) as reported by Shim *et al.* (2002 ▸); and open squares from Touloukian *et al.* (1975 ▸) as reported by Tsuchiya (2003 ▸). The red solid line represents our calculated real crystal model and the dashed black line indicates our perfect crystal model; for temperatures below about 800 K the heat capacity curves calculated from the two models are effectively identical.

The derived values of *C*_P_ for our data, including the effect of the thermal defects *C*_vac_, are plotted in Fig. 10 ▸ and are compared with experimental data taken from the literature (*e.g.* Anderson *et al.*, 1989 ▸; Shim *et al.*, 2002 ▸; Tsuchiya, 2003 ▸; Yokoo *et al.*, 2009 ▸); a remarkably good agreement between calculated and experimental values is observed even at the highest temperatures. However, Balcerzak *et al.* (2014 ▸) report calculated specific heat values that are different by up to 3% from the experimental results, where for *T* near *T*_m_, the calculated specific heat is slightly higher than the experimental one. The same tendency has been observed by Yokoo *et al.* (2009 ▸).

## 4. Conclusions   

We have experimentally determined the unit-cell volume of Au from 40 K up to the melting point by X-ray powder diffraction. Over the temperature range investigated, the behaviour of the material may be adequately described by a Grüneisen approximation to the zero-pressure equation of state representing the thermal expansion of the ‘perfect crystal’, combined with the thermodynamic theory of point defects to include the contributions from lattice defects at high temperatures (‘real crystal’). Au shows a nonlinear increase in thermal expansion prior to melting, which is likely to be a result of the thermally induced generation of point defects above 800 K. This takes the form of a smooth trend that departs from the Grüneisen–Debye model over a large temperature range, beginning at *T*/*T*_m_ ≃ 1000/1337 ≃ 0.75, which is very similar to the temperature range where deviations from linearity have been observed in the elastic moduli (Collard & McLellan, 1991 ▸).

## Supplementary Material

Crystal structure: contains datablock(s) MGO_AU_100K_RIETVELD_publ, MGO_AU_100K_RIETVELD_overall, MGO_AU_100K_RIETVELD_phase_1, MGO_AU_100K_RIETVELD_phase_2, MGO_AU_100K_RIETVELD_p_01. DOI: 10.1107/S1600576718002248/ks5577sup1.cif

Crystal structure: contains datablock(s) MGO_AU_25C_RIETVELD_publ, MGO_AU_25C_RIETVELD_overall, MGO_AU_25C_RIETVELD_phase_1, MGO_AU_25C_RIETVELD_phase_2, MGO_AU_25C_RIETVELD_p_01. DOI: 10.1107/S1600576718002248/ks5577sup2.cif

Crystal structure: contains datablock(s) MGO_AU_800C_RIETVELD_publ, MGO_AU_800C_RIETVELD_overall, MGO_AU_800C_RIETVELD_phase_1, MGO_AU_800C_RIETVELD_phase_2, MGO_AU_800C_RIETVELD_p_01. DOI: 10.1107/S1600576718002248/ks5577sup3.cif

Crystal structure: contains datablock(s) MGO_AU_1054C_RIETVEL_publ, MGO_AU_1054C_RIETVEL_overall, MGO_AU_1054C_RIETVEL_phase_1, MGO_AU_1054C_RIETVEL_phase_2, MGO_AU_1054C_RIETVEL_p_01. DOI: 10.1107/S1600576718002248/ks5577sup4.cif

Supplementary table. DOI: 10.1107/S1600576718002248/ks5577sup5.pdf

## Figures and Tables

**Table 1:** Volumetric thermal expansion parameters of gold

Polynomial model [equations (1)–(2)]		
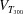 (Å^3^)	67.854 (2)	
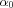 (K^−1^)	3.62 (2) × 10^−5^	
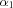 (K^−2^)	1.88 (3) × 10^−8^	

Second-order Grüneisen approximation [equation (3)]		
*Q* (J)	4.110 (8) × 10^−18^	
*b*	4.28 (4)	
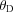 (K)	173 (2)	
*V*_0_ (Å^3^)	67.1657 (4)	

Derived values [equations (4) and (5), assuming γ = 2.95]		
*K*_0_ (GPa)	180.5 (4)	
*K*_0_′	9.57 (8)	

Third-order Grüneisen approximation [equation (6)]		
*Q* (J)	3.97 (2) × 10^−18^	
*b*	2.8 (2)	
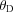 (K)	188 (2)	
*V*_0_ (Å^3^)	67.1678 (3)	
*c* (J^−1^)	−0.44 (5) × 10^19^	

Derived values [equations (4), (5) and (7) assuming γ = 2.95]		
*K*_0_ (GPa)	174.4 (9)	
*K*_0_′	6.6 (4)	
*K*_0_ *K*_0_′′	−100 (12)	
