# Supplementary material for: The thermal expansion of gold: point defect concentrations and pre-melting in a face-centred cubic metal
Source: J Appl Crystallogr. 2018 Mar 26;51(Pt 2):470–80. doi: 10.1107/S1600576718002248 (PMC5884389; doi:10.1107/S1600576718002248)
Supplement: Supplementary file 5 [file j-51-00470-sup5.pdf]

JOURNAL OF APPLIED CRYSTALLOGRAPHY

Volume 51 (2018)

Supporting information for article:

The thermal expansion of gold: point defect concentrations and pre-melting in an *fcc* metal

Martha G. Pamato, Ian G. Wood, David P. Dobson, Simon A. Hunt and Lidunka Vočadlo

Supplementary information

Table S1. Lattice parameter of gold as a function of temperature

Table S1. Lattice parameter of gold as a function of temperature

Temperature (K)	*a* (Å)	*V* (Å^3)
	Low Temperature stage	
40	4.06524(1)	67.1828(6)
50	4.06556(1)	67.1986(6)
60	4.06584(1)	67.2126(6)
70	4.06627(1)	67.2341(6)
80	4.06670(1)	67.2553(6)
90	4.06713(1)	67.2764(6)
100	4.06760(1)	67.2998(6)
110	4.06812(1)	67.3256(6)
120	4.06860(1)	67.3494(3)
130	4.06910(1)	67.3742(3)
140	4.06962(1)	67.4002(6)
150	4.07013(1)	67.4254(6)
160	4.07068(1)	67.4531(6)
170	4.07119(1)	67.4781(6)
180	4.07173(1)	67.5051(6)
190	4.07229(1)	67.5331(6)
200	4.07286(1)	67.5611(6)
220	4.07396(1)	67.6159(6)
240	4.07506(1)	67.6710(6)
260	4.07619(1)	67.7272(6)
280	4.07736(1)	67.7856(7)
300	4.07849(1)	67.8417(7)
	High Temperature stage	
298	4.07858(2)	67.8466(8)
313	4.07950(2)	67.8921(8)
333	4.08070(2)	67.9522(8)
353	4.08193(2)	68.0137(8)
373	4.08317(2)	68.0755(9)
393	4.08438(2)	68.1365(9)
413	4.08563(2)	68.1990(9)
433	4.08684(2)	68.2593(9)

453	4.08807(2)	68.3209(9)
473	4.08932(2)	68.384(1)
493	4.09055(2)	68.446(1)
513	4.09180(2)	68.508(1)
533	4.09305(2)	68.571(1)
553	4.09429(2)	68.634(1)
573	4.09558(2)	68.698(1)
593	4.09689(2)	68.764(1)
613	4.09812(2)	68.826(1)
633	4.09949(2)	68.895(1)
653	4.10074(2)	68.958(1)
673	4.10209(2)	69.0267(9)
693	4.10343(2)	69.0939(9)
713	4.10475(2)	69.1607(8)
733	4.10611(2)	69.2297(8)
753	4.10749(2)	69.2993(8)
773	4.10885(2)	69.3680(8)
793	4.11025(2)	69.4392(8)
813	4.11165(2)	69.5102(8)
833	4.11302(2)	69.5795(8)
853	4.11443(2)	69.6511(8)
873	4.11586(2)	69.7241(8)
893	4.11730(2)	69.7971(8)
913	4.11875(2)	69.8706(9)
933	4.12022(2)	69.9459(9)
953	4.12164(2)	70.0180(9)
973	4.12311(2)	70.0931(9)
993	4.12462(2)	70.170(1)
1013	4.12617(2)	70.249(1)
1033	4.12763(2)	70.324(1)
1053	4.12920(2)	70.404(1)
1073	4.13069(2)	70.480(1)
1093	4.13228(2)	70.562(1)
1113	4.13382(2)	70.641(1)
1133	4.13545(3)	70.724(1)
1153	4.13709(3)	70.809(1)
1173	4.13863(3)	70.887(1)
1193	4.14027(3)	70.972(1)
1213	4.14199(4)	71.061(2)
1233	4.14370(4)	71.148(2)
1253	4.14542(4)	71.237(2)
1273	4.14703(4)	71.320(2)
1283	4.14789(4)	71.364(2)
1293	4.14881(4)	71.412(2)
1303	4.14962(4)	71.454(2)
1313	4.15055(5)	71.502(2)

1315	4.15082(5)	71.515(2)
1317	4.15093(6)	71.521(3)
1319	4.15116(6)	71.533(3)
1321	4.15137(5)	71.544(3)
1323	4.15154(5)	71.553(2)
1325	4.15174(5)	71.563(2)
1327	4.15184(5)	71.569(2)
1329	4.15206(6)	71.580(3)
1331	4.15228(7)	71.591(4)
1333	4.15232(8)	71.593(4)
1335	4.1525(1)	71.604(5)

Values reported in the table represent unscaled data. Note, however, that in the subsequent analysis the unit-cell volumes determined in the high-temperature stage were scaled (by a factor of 0.99984) to match the low-temperature values (see text).
